# Effect of Life Satisfaction on Depression among Childless Married Couples: A Cross-Sectional Study

**DOI:** 10.3390/ijerph19042055

**Published:** 2022-02-12

**Authors:** Ju-Young Ha, Hyo-Jin Park

**Affiliations:** College of Nursing, Pusan National University, Yangsan 50612, Korea; jyha1028@pusan.ac.kr

**Keywords:** depression, personal satisfaction, family characteristics, actor-partner interdependence model, Korea

## Abstract

Depression among childless middle-aged and elderly people is a serious social problem in Korea. However, few studies examine the influence of life satisfaction on the depression of spouses as actors and partners. Hence, this study analyzes the influence of life satisfaction (a positive factor childless married couples may have) on depression. This cross-sectional study employed data on couples to analyze the effect of life satisfaction on the depression of childless married couples as actors and partners via the actor–partner interdependence model. The Korea Longitudinal Study of Aging was employed to investigate life satisfaction and depression among 207 childless middle-aged and elderly couples. Regarding actor effects, wives’ (β = −0.285, *p* = 0.004) and husbands’ (β = −0.403, *p* < 0.001) life satisfaction significantly affected individual depression. Regarding partner effects, husbands’ life satisfaction (β = −0.255, *p* = 0.011) significantly affected wives’ depression, and the wives’ life satisfaction (β = −0.375, *p* < 0.001) significantly affected husbands’ depression. A childless actor’s life satisfaction affected own and partner’s depression. Thus, spouses should work together to improve their life satisfaction, thereby improving their depression.

## 1. Background

The middle-aged are in their maturity stage of life, and the elderly can be said to be in their end-of-life stage. Such stages mark having completed raising children, developing and passing on cultural norms and values to the next generation, and acknowledging and reflecting on the meaning of life from the vast knowledge and experience gained [1]. In Korea, the middle-aged and elderly account for 55.4% of the population [2], with an aging rate that is the fastest worldwide. Moreover, the Confucian, patriarchal family system and respect for the elderly remain important in Korean society. Further, the situation of middle-aged and elderly groups who have experienced the effects of rapid industrialization and modernization is complex [3]. The elderly generation invested all their energy and resources into their children and social development [4]. Given the influence of family values, the family, directly and indirectly, influences individuals’ entire lives [5,6]. The more satisfactory a spousal relationship, the higher the psychological well-being [7] and the higher the quality of the parent–children relationship, the higher the life satisfaction [8].

Life satisfaction is one of the main indicators of quality of life as “a cognitive and judgmental process and a comprehensive assessment of human quality of life” [9]. Life satisfaction in middle age and old age, along with morale and psychological well-being, can be said to be an important indicator of successful adaptation to the aging process [10]. Medley [11] stated that life satisfaction is a subjective feeling of happiness and satisfaction for the whole of life and is a kind of attitude. He also said that successful aging is a concept that is complexly formed by individual values and self-concepts over a long period of time to feel satisfied and happy with life before old age and to lead a socially desirable life in old age.

In Korea, the relationship between parents and children is vital since social networks shrink in middle-age and elderly stages, and official duties disappear with retirement, leaving only the informal parenting role intact [12]. A study on the successful retirement scale development of the elderly in Korea identified “a life that is satisfied through child success” as a sub-factor [13]. Thus, Korean parents attach much meaning from how they relate to their children in evaluating their lives [14]. Further, rather than being simply members of a family, children are considered crucial in the cultural aspect of continuing the family line [15]. According to the results of empirical studies in Korea, regarding the elderly, the better the communication with adult children and the more consistent their values, the higher the life satisfaction [8]. Additionally, the greater the contact with children, the lower the feeling of depression [16] and the higher the life satisfaction [17].

In Korea, the middle-aged and elderly population group comprises 70% of depressed patients, with a high rate that increases with age [18]. Depression has a long-term impact on the quality of life of oneself and one’s family. Depression can lead to severe impairment in functioning and is associated with a high risk of suicide. Recently, rather than being regarded as a serious disease, depression has been emphasized as a mild disease that anyone can experience from time to time in daily life. In other words, depression is viewed as a disease with a kind of spectrum, and it is understood that it exists on a continuum from temporary emotional mood swings or normal sadness to pathologically severe depression [19]. If all cases with low-level depressive symptoms are included, the depression status of the middle-aged and elderly is even more serious.

In an empirical study on the influencing factors of depression in middle-aged and older people in Korea, family characteristics influence middle-age and elderly depression. Family characteristics influencing depression were spousal relationship, family support, and cohabitation with children. Low intimacy between couples and poor parent–children relationships increase loneliness and induce depression [20]. When a family member or spouses live together [21,22], with high support of spouse or child, depression is low [23]. Most studies on depression among the middle-aged and elderly examine the differences in the presence and absence of cohabitation with children, but few studies examine childless couples [20,21,22,23].

Spouses and children affect life satisfaction among the middle-aged and elderly. Moreover, individuals are influenced by spouses. Thus, the life satisfaction of childless couples can be evaluated simultaneously via the actor effect that affects an individual and the partner effect that affects a partner in the causal relationship with depression. However, prior studies on the relationship between life satisfaction, depression, and childlessness among the middle-aged and elderly mainly examine individuals [8,16,17,20,21,22,23].

Per the interdependence theory, couples closely influence each other daily and share various environmental factors and experiences [24]. Therefore, the actor–partner interdependence model (APIM) is useful to analyze the effect of life satisfaction on the depression of childless married couples and evaluate the bidirectional influence of the couples. The purpose of examining the effect of life satisfaction on depression of middle-aged and elderly couples in the absence of children is to successfully lead the aging process that middle-aged and elderly couples must go through together. Thus, this study aims to (a) evaluate whether there are differences in the level of life satisfaction and depression between male and female dyads of the childless middle-aged and elderly over 45 years old and (b) employ the APIM approach to elucidate and differentiate actor and partner effects of life satisfaction on depression.

## 2. Methods

### 2.1. Participants and Study Design

This study employs a cross-sectional approach via the APIM to analyze actor and partner effects of life satisfaction of childless middle-aged and elderly married couples on depression. It also employs data from the seventh Korea Longitudinal Study of Aging (KLoSA) by the Korea Employment Information Service. Out of 1684 middle-aged and elderly couples (excluding categories of divorce, separation, and bereavement), 207 without children were sampled for analyses. According to Song and Kim, the minimum recommended level for the sample size in the structural equation model is 10 times the free parameter, and the ideal recommended size is 150–400 people [25]. In addition, according to Ledermann et al., a sample size of about 93–241 couples was recommended in the Actor–Partner Interdependence Model [26]. Therefore, in this study, 207 couples (414 people) were selected as a sample, which is close to the recommended sample size.

### 2.2. Data Collection

KLoSA provides basic data to establish social and economic policies. Given the growing aging trend, KLoSA secures micro-data on the elderly, such as labor supply and retirement, income, consumption behavior, health, and social security system benefits, which are challenging to attain through cross-sectional surveys [27]. The population comprises citizens (excluding Jeju Island) over the age of 45. The sampling frame was the survey area of the 2005 Population and Housing Census, among which 261,237 ordinary and apartment (excluding island and facility unit) survey areas were set as the extraction unit survey area. The KLoSA survey was conducted in an even-numbered yearly trend from 2006, employing similar survey items as personal interviews. Employing the KLoSA’s raw data, questionnaire, and coding book for research means signing up as a member on the survey website (http://survey.keis.or.kr/ accessed on 5 November 2020) of the Korea Employment Information Service, disclosing the purpose of research, and agreeing to comply with the management regulations.

### 2.3. Instruments

This study employed some of the contents of the seventh KLoSA survey.

#### 2.3.1. Life Satisfaction

KLoSA gauged life satisfaction via the following questions on health status, economic condition, relationship with spouse, relationship with children, and overall quality of life: “How satisfied are you with your health?”; “How satisfied are you with your economic status?”; “How satisfied are you with your relationship with your spouse?”; “How satisfied are you with your relationship with your children?”; and “How satisfied are you with your overall quality of life (happiness) relative to other people of your age?”

This study excludes “How satisfied are you with your relationship with your children?” because it targets childless middle-aged and elderly couples. All four items were measured on a 10-point Likert scale, the average of which corresponded to life satisfaction level. Corresponding indicators ranged from 0 to 10 points; the higher the score, the higher the life satisfaction. The Cronbach’s α reliability coefficient value was 0.87.

#### 2.3.2. Depression

Depression was measured via the Center for Epidemiologic Studies Depression (CES-D) 10; the Korean CES-D 10 was abbreviated and employed in the US CES-D 20 items developed for chronically ill elderly patients. Out of 10 questions, 3 catered to depression; 2 to positive emotion; 3 to somatic symptom; and 2 to interpersonal relationship. Each question was measured on a four-point Likert scale; “I thought that kind of thing for a while, or I didn’t think that way (less than a day)” was scored 0; “I sometimes thought that way (about a day or two)” was scored 1; “I often thought that way (about 3 to 4 days)” was scored 2; and “I often thought that way (about 5 to 7 days)” was scored 3. The combined depression score ranges from 0 to 30; the higher the measurement score, the higher the degree of depression. Among the sub-areas, two positive emotions were reverse-coded and used as reverse scoring items. The Cronbach’s α reliability coefficient value was 0.88.

#### 2.3.3. Demographic Characteristics of the Couples

Data on the demographic characteristics of couples, such as age, education, perceived economic status, and economic activity, were obtained via the KLoSA survey.

### 2.4. Statistical Analysis

The data collected were analyzed using the Statistical Package for Social Sciences (SPSS) 22.0 and Analysis of Moment Structures (AMOS) 25.0. Participants’ demographic characteristics and couples’ measurement variables were presented via SPSS descriptive statistics to measure the skewness and kurtosis and test the data normality.

Moreover, the study employed Pearson’s correlation coefficients to check the correlation and multicollinearity of each factor and the measurement variables. It employed the AMOS structural equation model to identify the actor and partner effects of the life satisfaction of childless middle-aged and elderly couples on their depression. We chose the structural equation model because it can statistically compare and evaluate the magnitudes of the estimates obtained through model verification.

Further, confirmatory factor analysis (CFA) was conducted to investigate the validity of the latent variables for the model. We evaluated model fit based on a combined consideration of the goodness-of-fit and incremental fit indices. The former included chi-square test (χ^2^), χ^2^/df, root-mean-square error of approximation (RMSEA), standard root-mean-square residual (SRMR), goodness-of-fit index (GFI), and adjusted goodness-of-fit (AGFI). The latter included the incremental fit index, comparative fit index (CFI), normed fit index (NFI), incremental fit index (IFI), and Tucker–Lewis index (TLI). The χ^2^ should be as small as possible. A CFI value of 0.90 or higher indicates a reasonable fit, whereas an RMSEA value of 0.06 or lower and a SRMR value of 0.08 or lower indicate acceptable fit [28]. Finally, bootstrapping in AMOS was employed to verify the statistical significance of the paths in the structural equation model.

## 3. Results

### 3.1. Demographic Characteristics of Childless Middle-Aged and Elderly Couples

Table 1 presents the demographic characteristics of childless middle-aged and elderly couples. The average ages were 69.43 (66.19) years for husbands (wives). High school graduates were highest among husbands at 42.5%, followed by elementary school graduates and lower at 29.0% and middle school graduates, 16.9%. Among wives, 42.0% were elementary school graduates and lower, 34.3% were high school graduates, and 17.9% were middle school graduates. Among the couples participating in the study, 48.3% (33.3%) of husbands (wives) engaged in economic activities. Regarding their economic activity, most fell under dual-unemployed at 41.5%; single-income, 35.3%; and dual-income, 23.2%. On the subjective economic level, 52.7% (53.1%) of husbands (wives) answered “low.”

### 3.2. Life Satisfaction and Depression of Male and Female Dyads

As Table 2 shows, wives had lower life satisfaction than husbands (5.83 vs. 5.95; *p* = 0.012). Moreover, wives had higher depression than husbands (7.28 vs. 7.04; *p* < 0.001). Further, regarding the subscales of depression, wives had higher or similar scores than husbands in all domains of depression.

### 3.3. Confirmatory Factor Analysis

Before proceeding with the structural model analysis, confirmatory factor analysis (CFA) was performed to determine whether the observed variables constituting each latent variable were properly constructed. The results were as follows: χ^2^ = 66.942 (*p* < 0.001); degree of freedom (df) = 37; χ^2^/df = 1.762; NFI = 0.963; GFI = 0.950; AGFI = 0.900; CFI = 0.980; IFI = 0.982; TLI = 0.966; RMSEA = 0.061; and SRMR = 0.069. The value of the χ^2^/df index is ≤3. SRMR is acceptable if the value is ≤0.08 [29]. RMSEA, which considers both model error and simplicity simultaneously, is appropriate if it is ≤0.08. NFI, the standard GFI, is appropriate if it is ≥0.80. GFI and AGFI values are appropriate if higher than 0.90 [30]. Incremental fit indices, CFI, IFI, and TLI estimates are good if they are ≥0.9, being close to 1 [29].

### 3.4. Test of the Measurement Model

Before verifying the structural model, CFA via AMOS 25.0 was conducted to verify the validity of the questionnaire items and factors to be incorporated into the structural model. In addition, the convergent and discriminant validities were verified, as described below.

Standardized Factor Loadings (FL) > 0.70, Construct Reliability (CR) > 0.70, and Average Variance Extracted (AVE) > 0.50 between the questionnaire and factors verified the convergent validity [31]. Among the items to measure depression, the positive emotions of wives and husbands did not meet the criteria and were thus removed. That is, three sub-factors of depression for wives and husbands and four sub-factors of life satisfaction were used in the final analysis to verify conceptual reliability and average variance extraction. Thus, convergent validity was verified above the standard values of 0.70 and 0.50.

Next, this study verified whether the correlation coefficient between the factors was less than the square root of the AVE of each factor to verify the discriminant validity. Accordingly, the correlation coefficient of all factors was lower than the square root of AVE, verifying the discriminant validity.

### 3.5. Test of the Structural Model

Further, to find the actor and partner effects of couples’ life satisfaction on depression, the study evaluated the normality of the measured variables before modeling a structural equation. Moreover, to test the univariate normality of the measured variables, we calculated the skewness and kurtosis. The assumption of normal distribution was satisfied because the skewness was ≤3, and kurtosis was ≤10 for both husbands and wives. Additionally, as Table 3 shows, correlations ranged from −0.546 to 0.773.

The model has the following characteristics: χ^2^ = 66.942 (*p* < 0.001); df = 38; χ^2^/df = 1.762; NFI = 0.963; GFI = 0.950; AGFI = 0.900; CFI = 0.984; IFI = 0.984; TLI = 0.966; RMSEA = 0.061; and SRMR = 0.069. Thus, the model was found to have a good fit, as shown in Table 4.

### 3.6. Impact of Life Satisfaction on Depression at the Dyadic Level

**Figure 1:**
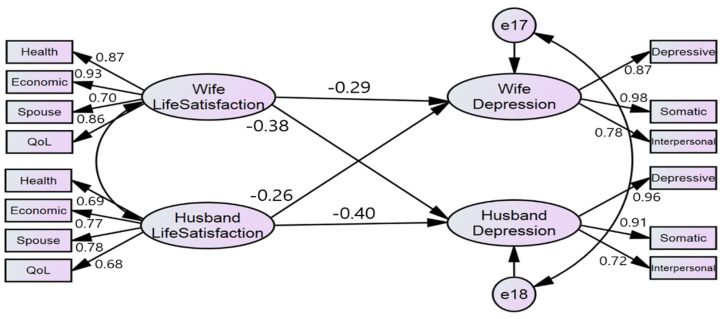
Assessment of the Hypothetical Model (the results of APIM for the dyadic effect of life satisfaction on depression).

Table 4 and Figure 1 shows the results of the actor and partner effects of couples’ life satisfaction on depression. The life satisfaction of wives (β = −0.285, *p* = 0.004) and husbands (β = −0.403, *p* < 0.001) had a significant effect on individual depression. Husbands’ life satisfaction (β = −0.255, *p* = 0.011) had a significant effect on wives’ depression, and wives’ life satisfaction (β = −0.375, *p* < 0.001) had a significant effect on husbands’ depression.

Regarding the effect on the depressive emotion domain, the life satisfaction of wives (β = −0.197, *p* = 0.007) and husbands (β = −0.401, *p* < 0.001) as actors significantly affected their depressive emotion domain. Regarding partner’s life satisfaction, husbands’ life satisfaction (β = −0.395, *p* < 0.001) significantly affected wives’ depressive emotion domain, and wives’ life satisfaction (β = −0.376, *p* < 0.001) significantly affected husbands’ depressive emotion domain.

Regarding the effect on the somatic symptom domain, the life satisfaction of wives (β = −0.554, *p* < 0.001) and husbands (β = −0.371, *p* < 0.001) as actors had a significant effect on their somatic symptom domain. As partners, husbands’ life satisfaction (β = −0.122, *p* = 0.030) significantly affected wives’ somatic symptom domain, whereas wives’ life satisfaction (β = −0.108, *p* = 0.142) had no significant effect on husbands’ somatic symptom domain.

Regarding the effect on the interpersonal relationship domain, the life satisfaction of wives (β = −0.566, *p* < 0.001) and husbands (β = −0.339, *p* < 0.001) as actors significantly affected their interpersonal relationship domain. As partners, husbands’ life satisfaction (β = −0.382, *p* < 0.001) significantly affected wives’ interpersonal relationship domain, whereas wives’ life satisfaction (β = −0.096, *p* = 0.168) had no significant effect on husbands’ interpersonal relationship domain.

This study examines whether the actor and the partner effects differed between men and women; the two coefficients were considered equal and compared using the chi-square test for the constrained and unconstrained (saturated) models (Table 5). No significant difference was noted between the constrained model, where both the husband and wife’s actor effects were constrained equally, and the unconstrained model (χ^2^ = 0.344, *p* = 0.557). This result reveals that the life satisfaction effect of husband and wife actors had a similar impact on depression. However, there was a significant difference between the constrained model, where the partner effects of husbands and wives were constrained equally, and the unconstrained model (χ^2^ = 9.455, *p* = 0.002). The findings indicate that the life satisfaction partner effect of husbands significantly impacted depression more than that of wives. Further, there was a significant difference in the equivalence constraints between the actor and partner effects for husbands’ life satisfaction (χ^2^ = 6.360, *p* = 0.030), and that of wives had no significant difference. (χ^2^ = 2.988, *p* = 0.084). This result reveals that the partner effect of husbands’ life satisfaction on depression was more influential than that of wives.

## 4. Discussion

This study aims to understand the actor and partner effects of the life satisfaction of childless middle-aged and elderly couples on depression using data from the seventh KLoSA in 2018. While most prior studies on the middle-aged and elderly regarding the relationship with children, life satisfaction, and depression have been fragmentary or individual studies [8,16,17,20,21,22,23], this study is the first to assess the relationship between life satisfaction and depression among middle-aged and elderly couples using the APIM approach.

Confucian culture, one of the traditional ideologies of Korea, emphasizes the filial responsibility of children to respect and support their parents. In particular, it emphasizes the meaning of having children and passing on the family, and there is a tendency to view the relationship between parents and children as the center of family relationships. In this traditional thought, the absence of children is not seen as a complete family. They also tend to feel guilty about not being able to carry on the family line [3].

For middle-aged and elderly couples, the stronger the emotional bond with their children, the higher their life satisfaction and the lower their depression. In addition, positive bonding with children is shown to strengthen individual self-esteem in middle-aged and elderly couples [20,21,22,23]. That is, in the case of middle-aged couples who have a positive emotional bond with their children, they have a higher will to solve problems and a higher will to achieve personal well-being by strengthening their personal self-esteem. As a result, life satisfaction is high, and depression is low, indicating that children are very special to middle-aged and elderly couples [32].

Neugarten, Harvighurst, and Tobin [33] defined life satisfaction as having a positive self-image and maintaining a positive attitude toward one’s life at the same time as feeling pleasure in activities that constitute daily life. Life satisfaction in middle age and old age in the process of aging is highlighted as an important factor along with objective life satisfaction and subjective life satisfaction [34]. In other words, it is because one feels a sense of psychological well-being or happiness by acknowledging how valuable and successful one’s life was in judging oneself. Depression can be cited as a factor that has the greatest influence on life satisfaction in middle-aged and elderly people, and it was found that the depressed elderly had lower life satisfaction [35].

Depression in the elderly is often accompanied by other health problems or life events, and most of the depressive symptoms are considered a natural reaction due to aging and are often neglected or overlooked. Depression in middle age and old age tends to consist of more depressive symptoms as the number of health problems increases, and life events that cause depression include job loss, retirement, death of a spouse, divorce, and loss of economic power [36]. The main factors for depression among the elderly in Korea were family factors, such as marital intimacy, cohabitation with children, relationships with children, and family support [37]. There were differences in depression according to these family factors. Depression is an important variable as an indicator of the psychological and mental health of the elderly. Older people generally tend to think more negatively about reality related to themselves than they is actually true, and in particular, depressed elderly people tend to interpret their lives with negative attitudes, such as black-and-white logic, and view themselves as failures. It is necessary to help them to accept aging positively [38].

The effectiveness of programs focusing on positive emotions as a way to overcome depression in middle age and old age has been proven [39,40,41]. Reinforcing positive self-awareness and positive thinking is useful in depression intervention. In addition, as parameters affecting overcoming depression, there are support systems, such as social support and family support [21,22,23,42]. If there is a support system that provides psychological support, the ability to overcome depression is better. In other words, in order to reduce depression in middle age and old age, it is possible to overcome depression more effectively if we find the positive strengths and personality strengths of each individual, give meaning to life, and use the psychological support system. Therefore, in order to overcome depression in couples without children, it is necessary to check the effects of one’s positive emotions on oneself and one’s spouse and the effects of one’s spouse’s positive emotions on oneself. This study tried to confirm the effect of life satisfaction as a positive emotion to overcome depression in middle-aged couples without children. In addition, the spouse effect was confirmed using the APIM model to prepare a plan to utilize the spouse’s support system to reduce depression.

In this study, life satisfaction and depression of middle-aged and elderly couples without children were analyzed. Compared with the results of analysis of life satisfaction and depression of all middle-aged and older people using KLoSA in a study by Shu et al. [43], the life satisfaction of middle-aged and elderly couples without children was lower than the average life satisfaction of general middle-aged and elderly couples. Furthermore, the depression of middle-aged and elderly couples without children was higher than the average depression of general middle-aged and elderly couples. These results are consistent with the research findings that the better the relationship with children and the more frequent they meet, the higher the life satisfaction and the lower the loneliness and depression [16,17,20,21,22,23]. In particular, the life satisfaction and depression of middle-aged and elderly couples without children were found to be lower in the wife’s life satisfaction and higher in wife’s depression than in the husband. This is consistent with the results of Kim and Park’s study that the frequency of depression according to gender was significantly higher in the female elderly than in the male elderly [23]. In addition, as a variable on loneliness and depression, it was found that the influence of the relationship with children had a greater effect on the female elderly than the male elderly [20]. Therefore, in the case of a middle-aged and elderly couple without children, both husband and wife had low life satisfaction and high depression, and among couples, especially the wife had low life satisfaction and high depression.

After examining the actor effects of life satisfaction on depression, wives and husbands had actor effects on depression. That is, their life satisfaction significantly affected their depression. These results follow prior studies that verified the negative relationship between life satisfaction and depression in middle-aged or elderly individuals [44,45,46,47]. Thus, this study indicates that it is essential to improve life satisfaction to lower the depression of childless middle-aged and elderly couples. Particularly, after examining the equivalence constraint model to determine the magnitude of the actor effect of wives and husbands on depression, there was no significant difference. Hence, the life satisfaction of wives and husbands was confirmed to affect their depression reduction.

Further, after examining the partner effect of life satisfaction on depression, the life satisfaction of wives and husbands had a partner effect on depression. That is, the life satisfaction of partners showed a significant effect on the depression of wives and husbands. These results were consistent with prior studies [48,49,50], where depression was related to the difference in spousal influence. Moreover, it is in the same context as the result where the similarity of depression between couples persists over time by measuring the depressive change of couples over 10 years with 905 couples in Belgium via the latent growth model [51].

However, based on the test of the equivalence constraint model on the partner effect, the size of the partner effect model of wives and the husbands was different in the effect of life satisfaction on depression. In addition, the actor effect model of wives’ life satisfaction and that of husbands generated no significant difference. Moreover, the actor effect model of husbands’ life satisfaction and that of wives yielded a significant difference. In other words, the test of the equivalence constraint model is summarized as follows: There was no significant difference between the husband’s actor effects and the wife’s partner effects (for husbands, their life satisfaction affects their depression. Wife’s life satisfaction influences her own depression. There is no significant difference between these two effects). There was a significant difference between the husband’s partner effects and the wife’s partner effects (a husband’s life satisfaction affects wife’s depression. A wife’s life satisfaction affects her husband’s depression. There is a significant difference between these two effects. The effect size of husband on wife was larger than the effect of a wife on husband). There was a significant difference between the husband’s actor effects and the wife’s partner effects (husband’s life satisfaction affects his own depression. A wife’s life satisfaction affects husband’s depression. There is a significant difference between these two effects. For husbands, the effect size of own life satisfaction on their depression was larger than the effect size of the wife’s life satisfaction on their depression). There was no significant difference between the wife’s actor effects and the husband’s partner effects (wife’s satisfaction with her own life affects her depression. The husband’s life satisfaction affects the wife’s depression. There is no significant difference between these two effects).

The difference in the partner effects between wives and husbands can be interpreted regarding the difference in empathy per the gender and emotional contagion between couples. According to Hoffman [52], empathic ability is an emotional response to that of others; women showed a higher level of empathy than men. Given that depressive symptoms are feelings of sadness, depression, and droopiness over time as well as a loss of interest or pleasure in daily activities [53], wives are more concerned with the feelings and moods of spouses than are husbands. Having much empathy may affect depression symptoms. Similarly, emotional contagion induces a similar emotion in a person nearby; reportedly, women are more sensitive to the emotions of others than are men [54]. Prior studies on emotional contagion mainly examine the transfer of depression; reportedly, such transmission did not significantly affect the level of depression in husbands [48,55,56]. This study confirmed that life satisfaction of childless middle-aged and elderly couples affected the depression of others; the partner effect of wives and husbands yielded a difference in magnitude. Further research can determine whether the emotional contagion of depression between husband and wife is significant.

Given that this study employed cross-sectional collection data to analyze middle-aged and elderly couples, the generational differences of childless couples could not be identified. Further, there are limitations in explaining causal relationships. Therefore, future studies can investigate the causal relationship between variables through longitudinal data analysis and compare and analyze the generations of childless couples. In addition, there is a limitation in that only life satisfaction was examined as affecting the depression of middle-aged and elderly couples without children. Therefore, studies on the effects of various factors affecting depression in childless couples and variables that can overcome them should be conducted in the future.

In summary, beyond the correlation between life satisfaction and depression of childless middle-aged and elderly couples, actor and partner effects were clearly confirmed. That is, given that the life satisfaction and depression of husbands and wives affect each other, childless middle-aged and elderly couples must be recognized as non-independent beings with mutual relationships rather than individual beings. In addition, in the intervention study to reduce their depression, couples must participate together and actively seek ways to resolve depression by enhancing their life satisfaction.

The study findings suggest the following areas for future research. First, it is necessary to develop a program for childless middle-aged and elderly couples to participate together and increase their life satisfaction, as the life satisfaction of childless middle-aged and elderly couples positively affects their own depression and that of their partners. Second, it is necessary to analyze the interactions on life satisfaction and depression of childless middle-aged and elderly couples in various cultures using APIM models. The generation of middle-aged and elderly couples in Korea values the tradition of a Confucian and patriarchal family system. Thus, family values relative to different cultures can be compared. Third, it is necessary to compare and analyze life satisfaction and depression of voluntary childless couples and middle-aged and elderly couples. The DINK phenomenon is occurring among dual-income couples of the younger generation. It describes the situation where people voluntarily choose to have no children for economic stability. However, there is a limitation in that the childless middle-aged and elderly couple analyzed in this study were not investigated as to whether they were involuntarily or voluntarily childless. Thus, it is necessary to examine life satisfaction and depression relative to middle-aged and elderly couples in the involuntarily childless group.

## 5. Conclusions

This study employed the APIM approach to identify the effect of life satisfaction of childless middle-aged and elderly couples on depression from actor and partner perspectives. The results showed that the life satisfaction of childless middle-aged and elderly couples affected their depression both as actors and partners. This study found that spouses mutually influence each other regarding life satisfaction and depression; thus, it is necessary to develop intervention programs involving couple (not just individual) participation, where they learn to impact each other positively. Regarding the actor effect of life satisfaction, there was no significant difference between husbands and wives, as they affected each other’s depression. However, regarding the partner effect of life satisfaction, the degree to which the husbands and wives affected depression differed. Both couples were affected by the partner effect; even so, the partner effect of husbands was greater than that of wives.

Therefore, it is necessary to improve the life satisfaction of the couple to curb the depression of childless middle-aged and elderly couples. In particular, both the actor and partner effects should be employed to control wives’ depression. Further, to control husbands’ depression, improving one’s own life satisfaction should be prioritized.

## Figures and Tables

**Table 1:** Demographic Characteristics of Couples (*n* = 207 couples).

		Couples	Wives	Husbands
		N (%)	N (%)	N (%)
Age (years)	Range		57–90	57–91
	≤64		101 (48.8)	68 (32.9)
	65–74		69 (33.4)	85 (41.1)
	75–84		33 (15.9)	46 (22.2)
	≥85		4 (1.9)	8 (3.8)
	≤Elementary		87 (42.0)	60 (29.0)
Education	Middle		37 (17.9)	35 (16.9)
	High		71 (34.3)	88 (42.5)
	≥College		12 (5.8)	24 (11.6)
	High		3 (1.4)	5 (2.4)
Perceived economic status	Medium		94 (45.4)	93 (44.9)
	Low		110 (53.1)	109 (52.7)
	Yes		69 (33.3)	100 (48.3)
Economic activity	No		138 (66.7)	107 (51.7)
	Dual-income	48 (23.2)		
	Single-income	73 (35.3)		
	Dual-unemployed	86 (41.5)		

**Table 2:** Level of Life Satisfaction and Depression among Childless Middle-aged and Elderly couples (*n* = 207 couples).

Variables	Wives M ± SD	Husbands M ± SD	*t* Value	*p*-Value
Life satisfaction	5.83 ± 1.45	5.95 ± 1.46	5.94	0.012
Health state	5.38 ± 1.81	5.49 ± 1.97	−2.07	0.039
Economic state	5.37 ± 1.76	5.33 ± 1.80	−1.04	0.069
Spouse satisfaction	6.41 ± 1.77	6.77 ± 1.75	6.65	<0.001
Overall quality of life	6.14 ± 1.74	6.20 ± 1.69	−8.75	<0.001
Depression	7.28 ± 6.05	7.04 ± 6.48	−1.43	<0.001
Depressive emotion	1.76 ± 2.13	1.70 ± 2.41	−8.57	<0.001
Positive emotion	2.65 ± 2.04	2.48 ± 1.98	−12.50	<0.001
Somatic symptom	1.86 ± 2.21	1.84 ± 2.47	−1.41	0.095
Interpersonal relationship	1.01 ± 1.42	1.02 ± 1.45	−6.24	<0.001

Note. M ± SD = mean ± standard deviation.

**Table 3:** Correlation Between Life Satisfaction and Depression among Childless Middle-aged and Elderly Couples (*n* = 207 couples).

	1	2	3	4	5	6	7	8	9	10	11	12	13	14	15	16
Wives’ life satisfaction																
1 Health state	1															
2 Economic state	0.59 **	1														
3 Spouse satisfaction	0.51 **	0.52 **	1													
4 Overall quality of life	0.51 **	0.57 **	0.62 **	1												
Wives’ depression																
5 Depressive emotion	−0.46 **	−0.28 **	−0.52 **	−0.37 **	1											
6 Positive emotion	0.27 **	0.11	0.14 *	0.28 **	−0.18 *	1										
7 Somatic symptom	−0.51 **	−0.34 **	−0.47 **	−0.35 **	0.76 **	−0.19 **	1									
8 Interpersonal relationship	−0.36 **	−0.26 **	−0.43 **	−0.25 **	0.71 **	−0.22 **	0.68 **	1								
Husbands’ life satisfaction																
9 Health state	0.51 **	0.47 **	0.32 **	0.41 **	−0.44 **	0.10	−0.51 **	−0.44 **	1							
10 Economic state	0.44 **	0.60 **	0.36 **	0.51 **	−0.25 **	0.24 **	−0.32 **	−0.21 **	0.63 **	1						
11 Spouse satisfaction	0.35 **	0.34 **	0.56 **	0.40 **	−0.41 **	0.22 **	−0.40 **	−0.38 **	0.42 **	0.40 **	1					
12 Overall quality of life	0.44 **	0.49 **	0.47 **	0.58 **	−0.39 **	0.14 *	−0.46 **	−0.34 **	0.57 **	0.62 **	0.58 **	1				
Husbands’ depression																
13 Depressive emotion	−0.41 **	−0.31 **	−0.44 **	−0.30 **	0.71 **	−0.27 **	0.70 **	0.63 **	−0.54 **	−0.29 **	−0.39 **	−0.42 **	1			
14 Positive emotion	0.28 **	0.16 *	0.14 *	0.15 *	−0.05	0.73 **	−0.05	−0.06	0.01	0.08	0.22 **	0.16 *	−0.05	1		
15 Somatic symptom	−0.40 **	−0.38 **	−0.40 **	−0.32 **	0.65 **	−0.21 **	0.69 **	0.57 **	−0.55 **	−0.34 **	−0.32 **	−0.40 **	0.77 **	−0.01	1	
16 Interpersonal relationship	−0.38 **	−0.24 **	−0.36 **	−0.28 **	0.57 **	−0.25 **	0.62 **	0.69 **	−0.42 **	−0.19 **	−0.33 **	−0.27 **	0.69 **	−0.04	0.64 **	1

*p*-value: * *p* < 0.05, ** *p* < 0.01.

**Table 4:** Effect Coefficients for the Hypothetical Model (*n* = 207 couples).

		Wives			Husbands		
		Β (SE)	*t* Value	*p*-Value	Β (SE)	*t* Value	*p*-Value
Total depression	Actor’s life satisfaction	−0.285(0.046)	2.889	0.004	−0.403(0.025)	3.880	<0.001
	Partner’s life satisfaction	−0.375(0.038)	3.766	<0.001	−0.255(0.029)	2.530	0.011
Depressive emotion	Actor’s life satisfaction	−0.197(0.062)	1.449	0.007	−0.401(0.026)	3.420	<0.001
	Partner’s life satisfaction	−0.395(0.021)	3.890	<0.001	−0.376(0.021)	3.877	<0.001
Somatic symptom	Actor’s life satisfaction	−0.554(0.029)	5.074	<0.001	−0.371(0.037)	2.267	<0.001
	Partner’s life satisfaction	−0.122(0.051)	1.377	0.030	−0.108(0.064)	1.382	0.142
Interpersonal relationship	Actor’s life satisfaction	−0.566(0.027)	5.102	<0.001	−0.339(0.040)	1.178	<0.001
	Partner’s life satisfaction	−0.382(0.029)	3.857	<0.001	−0.096(0.071)	1.215	0.168

Note. SE, standard error.

**Table 5:** χ^2^ Differences in the Test Between the Basic and Equivalent Constraint Models.

Model	χ2	df	TLI	CFI	RMSEA	Δχ2	*p*-Value
basic	66.942	38	0.966	0.984	0.061		
equivalence constraint 1 (a = b)	67.286	39	0.973	0.984	0.059	0.344	0.557
equivalence constraint 2 (a1 = b1)	76.397	39	0.972	0.984	0.060	9.455	0.002
equivalence constraint 3 (a = b1)	73.302	39	0.970	0.983	0.062	6.360	0.030
equivalence constraint 4 (b = a1)	69.930	39	0.970	0.982	0.062	2.988	0.084

Note. CFI, Comparative Fit Index; df, degree of freedom; RMSEA, Root Mean Squared Error of Approximation; TLI, Tucker–Lewis Index; a, husband’s actor effects; b, wives’ actor effects; a1, husbands’ partner effects; b1, wives’ partner effects.

## Data Availability

All data generated or analyzed during this study are included in the published article. For further clarifications, the authors can be contacted at hyojin@pusan.ac.kr.

## Abbreviations

KLoSA	Korea Longitudinal Study of Aging
APIM	Actor–partner interdependence model
(χ^2^), χ^2^/df	Chi-square
RMSEA	Root-mean-square error of approximation
SRMR	Standardized root-mean-square residual
GFI	Goodness-of-fit index
AGFI	Adjusted goodness-of-fit index
CFI	Comparative fit index
NFI	Normed fit index
IFI	Incremental fit index
TLI	Tucker–Lewis index
AMOS	Analysis of moment structures
SPSS	Statistical Package for the Social Sciences
DINK	Double Income, No Kids
